# Case Report: *MYH9*-related disease caused by Ala44Pro mutation in a child with a previous diagnosis of chronic immune thrombocytopenia

**DOI:** 10.3389/fped.2024.1391742

**Published:** 2024-05-17

**Authors:** Kaori Niwa, Hidemi Toyoda, Atsushi Kohso, Yosuke Okumura, Shinji Kunishima, Masahiro Hirayama

**Affiliations:** ^1^ Department of Pediatrics, Mie University Graduate School of Medicine, Tsu, Japan; ^2^ Department of Medical Technology, Gifu University of Medical Science, Gifu, Japan

**Keywords:** *MYH9*-related disease, chronic immune thrombocytopenia, macrothrombocytopenia, Döhle-like body, p.Ala44Pro mutation

## Abstract

*MYH9*-related disease, a rare autosomal dominant platelet disorder characterized by thrombocytopenia, giant platelets, and leukocyte inclusion bodies, may mimic immune thrombocytopenia in children unless suspected and carefully excluded. Here, we present a case involving a three-year-old girl with mild bleeding symptoms since infancy, previously diagnosed with chronic immune thrombocytopenia. The patient exhibited isolated thrombocytopenia and lacked any family history of thrombocytopenia, hearing impairment, or renal failure. Examination of peripheral blood smears via light microscopy revealed significant platelet macrocytosis with giant platelets and basophilic Döhle-like bodies in the neutrophils. Subsequent sequencing analysis of *MYH9* gene identified a p.Ala44Pro mutation. Throughout a six-year follow-up period, the patient's condition remained stable. Our report underscores the significance of identifying leukocyte inclusion bodies in peripheral blood smears and considering *MYH9*-related diseases, even in instances of chronic macrothrombocytopenia devoid of familial history or non-hematological manifestations.

## Introduction

Immune thrombocytopenia (ITP) is a common childhood bleeding disorder diagnosed by exclusion when isolated thrombocytopenia is not part of another disease process (1). In most cases, medical history, physical examination, and a complete blood count are sufficient to exclude secondary thrombocytopenia (e.g., multisystem autoimmune diseases, lymphoproliferative diseases, drug-induced thrombocytopenia, infections, and myelodysplastic syndromes). However, it is challenging to exclude inherited thrombocytopenia, such as *MYH9*-related disease (*MYH9*-RD), Bernard-Soulier syndrome (BSS), gray platelet syndrome, and von Willebrand disease type IIB, due to the rarity of these conditions and lack of awareness, leading to inappropriate treatment and delayed identification of the underlying condition. Clinical presentations of *MYH9*-RD typically include mild bleeding tendencies, easy bruising, epistaxis, menorrhagia in women, and postoperative hemorrhage, which depend on the severity of thrombocytopenia. However, some patients remain asymptomatic. *MYH9*-RD is occasionally discovered accidentally during routine blood tests in asymptomatic individuals. *MYH9*-RD is a frequent form of inherited thrombocytopenia and encompasses four autosomal-dominant thrombocytopenias that were previously described as distinct disorders, namely May-Hegglin anomaly (MHA), Fechtner syndrome, Sebastian syndrome, and Epstein syndrome (2). These syndromes are caused by mutations in the *MYH9* gene, which encodes non-muscle myosin heavy chain IIA (NMMHC-IIA) (3). Each NMMHC-IIA comprises three domains: head, neck, and tail. The human *MYH9* gene contains 41 exons spanning 33,320 bases and is located on chromosome 22 q12–13. Approximately 80 mutations, mostly point mutations, have been reported in *MYH9* in several pedigrees (4). Abnormal NMMHC-IIA expression may disrupt the composition and reorganization of the cytoskeleton, leading to abnormal platelet formation by megakaryocytes, resulting in macrothrombocytopenia (5–7). Characteristic clinical features include thrombocytopenia with giant platelets and polymorphonuclear Döhle-like bodies. Patients with *MYH9*-RD may develop non-hematological manifestations, including sensorineural deafness, nephropathy, and cataracts. Interestingly, an analysis of a large case series of patients has concluded that the clinical picture varied within families carrying the same mutation and among individual patients during their lifetime (8).

Here, we present a case of an exon 2 mutation c.130 G > C (p.Ala44Pro) in the *MYH9* gene in a patient with macrothrombocytopenia and mild bleeding symptoms in the absence of nephritis, cataracts, and deafness, who had been misdiagnosed with chronic ITP.

## Case description

The patient, born at term following an uncomplicated pregnancy, is the only daughter of healthy, non-consanguineous Japanese parents. There is no family history of hearing loss, renal insufficiency, presenile cataracts, or hematological disorders. Although she has had petechiae since infancy, she has not undergone any examinations. At the age of three years, thrombocytopenia was incidentally noted during a review for a generalized erythematous maculopapular rash. She was diagnosed with ITP and was subsequently followed up without treatment at a local hospital. At the request of the patient's family for an assessment of thrombocytopenia by pediatric hematologists, the patient was referred to our hospital.

**Figure 1:**
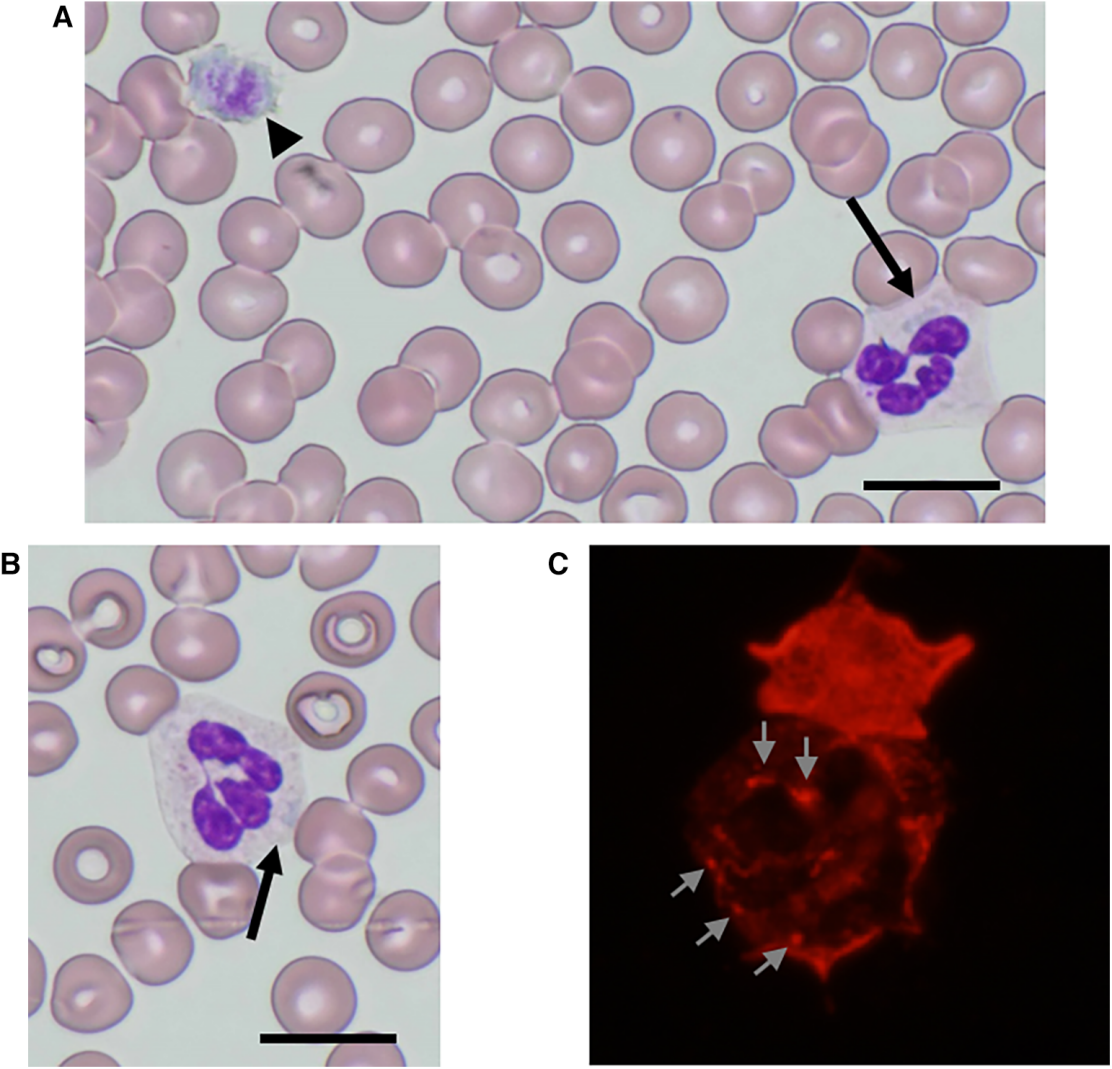
NMMHC-IIA localization in neutrophils from the patient. (**A,B**) May-Grünwald-Giemsa-stained peripheral blood smear images of the patient. Döhle-like body (arrow) and giant platelet as large as red blood cells (arrowhead) (**A**) Döhle-like body (arrow). Scale bars = 10 µm (**B**). (**C**) An immunofluorescence micrograph of a neutrophil immunostained with anti-NMMHC-IIA antibody. Small aggregates (<0.7 µm) with less than 11 in number are shown (arrows).

Physical examination revealed no purpura on the legs, petechiae in the oral cavity, or hearing loss. Peripheral blood analysis revealed thrombocytopenia, with a platelet count of 53 × 10^9^/L (normal range 150–400 × 10^9^/L). White blood cell and erythrocyte counts were 9.8 × 10^9^/L (normal range 4.0–15.0 × 10^9^/L) and 4.85 × 10^12^/L (normal range 4.00–5.30 × 10^12^/L), respectively. Other laboratory data, including creatinine, blood urea nitrogen, liver profiles, prothrombin time, and partial thromboplastin time, were all within normal limits, with a creatinine level of 0.23 mg/dl (normal range 0.20–0.39 mg/dl), an alanine aminotransferase level of 9 U/L (normal range 9–30 U/L), and an aspartate aminotransferase level of 62 U/L (normal range 24–44 U/L). Urinalysis results were normal, with no evidence of hematuria or proteinuria. Audiometric and ophthalmological findings were normal. Examination of peripheral blood films (May-Grünwald-Giemsa stain) by light microscopy showed giant platelets and Döhle-like inclusions in neutrophils (Figures 1A,B). Immunofluorescence analysis (IFA) for the NMMHC-IIA revealed small aggregates (<0.7 µm), less than 11 in number, predicting mutations in motor domain of *MYH9* gene (9) (Figure 1C). Bone marrow examination revealed an increased number of megakaryocytes without dysplasia. The presence of macrothrombocytopenia and Döhle-like bodies in neutrophils suggested a molecular investigation of the suspected *MYH9* mutations. The patient carried a previously described heterozygous transversion, c.130 G > C (p.Ala44Pro), in *MYH9* exon 2, which was diagnosed as *MYH9*-RD. Family members were excluded if they refused to participate. The segregation analysis could not be performed because the other family members did not consent to genetic testing.

No treatment was administered as no bleeding complications were observed. She has been in good condition for the last six years following diagnosis, without progression of thrombocytopenia, hearing impairment, renal insufficiency, or cataracts.

## Discussion

Although *MYH9*-RD, caused by a mutation of the *MYH9* gene, is the most common cause of inherited macrothrombocytopenia, it is a rare disorder in real-life scenarios. This rarity may lead to patients being misdiagnosed with more frequent bleeding disorders. Misdiagnosis of *MYH9*-RD as ITP is common due to overlapping clinical features, resulting in unnecessary diagnostic procedures and inappropriate treatments, such as undue immunosuppressive therapies and splenectomy. Approximately 60% of cases among patients enrolled in the Italian registry were initially diagnosed with ITP, with 30% and 12% receiving inappropriate treatments and unnecessary splenectomy, respectively (5). This highlights the necessity for a comprehensive approach, including a detailed family history and a careful review of peripheral blood films. Our patient was easily overlooked because she presented with subtle clinical characteristics, and a complete family history was not readily available.

The diagnosis of *MYH9*-RD has conventionally been based on morphological examination revealing a triad of giant platelets, thrombocytopenia, and Döhle-like bodies, along with inclusions in the cytoplasm of granulocytes in blood films. An extreme degree of platelet macrocytosis is commonly accepted as a hallmark of *MYH9*-RD and a crucial clue for identifying these patients (2, 5). A mean platelet diameter >3.7 µm and/or >40% of platelets larger than 3.9 µm show good sensitivity and specificity in distinguishing *MYH9*-RD from other forms of inherited or acquired thrombocytopenia (10). However, routine automated cell counters, utilizing impedenziometric or optical technology, primarily recognize platelets based on their size and often misclassify giant platelets of *MYH9*-RD as erythrocytes (2, 5). Consequently, these instruments overestimate the degree of thrombocytopenia and platelet macrocytosis (11). Therefore, microscopic or flow cytometric counting of platelets is important for the accurate measurement of platelet count in individuals with *MYH9*-RD, and microscopic examination of blood smears is essential for identifying prominent platelet macrocytosis (3). Döhle-like bodies, with a major diameter ranging from 1 to 2 µm, may be identified as faint, light-blue inclusions with different shapes (round, oval, or spindle-shaped) in the cytoplasm of neutrophils after conventional May-Grünwald-Giemsa staining of blood smears (12). Although they are identified in 42%–84% of patients with *MYH9*-RD and detected in 15%–100% of neutrophils, the difficulty in detecting inclusion bodies sometimes leads to the misdiagnosis of this disorder. Döhle-like bodies, the cytoplasmic aggregates of the NMMNC-IIA protein, can be detected in all neutrophils of patients with *MYH9*-RD after immunolabeling for NMMHC-IIA, and this assay has near 100% specificity and sensitivity for diagnosis (9, 12–15). Hao et al. applied a classification tree approach to hierarchically classify neutrophil NMMHC-IIA localization based on the size and number of NMMHC-IIA aggregates (9). Our patient had small aggregates (<0.7 µm) with less than 11 in number, predicting p.Arg702Cys or p.Ser96Leu mutations in motor domain (9). Genetic analysis of motor domain revealed p.Ala44Pro mutation in *MYH9* exon 2. Therefore, an IFA for NMMHC-IIA in peripheral blood smears should be performed as a diagnostic test for *MYH9*-RD.

**Figure 2:**
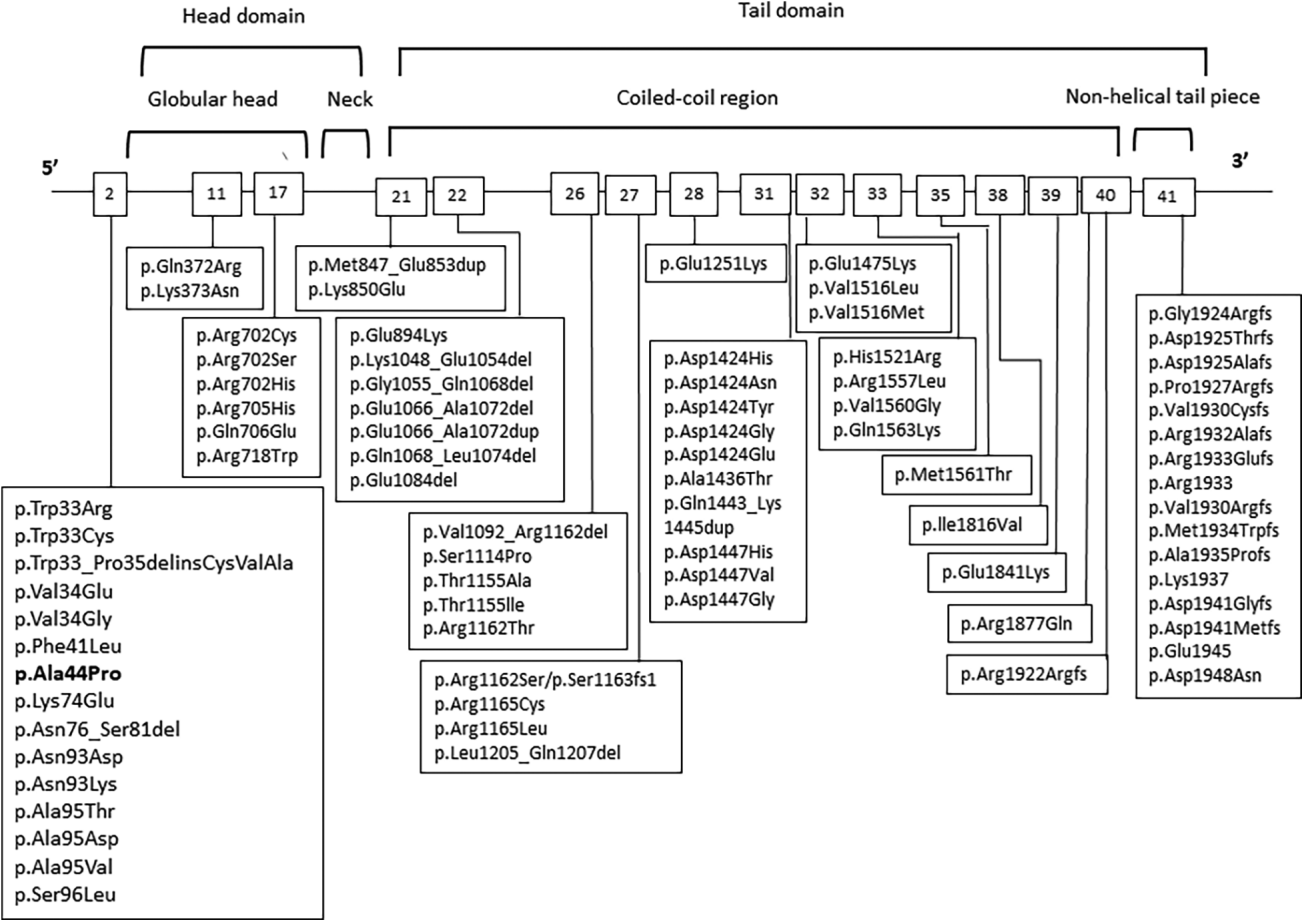
*MYH9* genetic variants in patients with *MYH9*-RD. Affected exons and mutations are shown.

The phenotype of patients with *MYH9*-RD can evolve, as supported by previous studies. Progressive manifestations that can develop over time include deafness, presenile cataracts, and nephropathy, which may ultimately progress to end-stage renal disease. Although the diagnosis of *MYH9*-RD can be confirmed by immunofluorescence assay for NMMHC-IIA using peripheral blood smears and identifying the causative *MYH9* mutation is not strictly required, it is important for providing prognostic assessment. A genotype-phenotype correlation has been recognized in *MYH9*-RD (16, 17). A higher incidence and worse prognosis of kidney impairment and deafness are associated with mutations affecting the head domain (exons 2–19) of NMMHC-IIA than with mutations in the tail domain (exons 20–41) (Figure 2) (2). Mutations of head domain were mainly located in two specific regions, the SH1 helix and the interface between the SH3-like motif and the motor domain (MD) (SH3/MD interface) (18, 19). Substitutions in the SH1 helix (corresponding to R702 substitutions) were associated with a much higher risk of nephropathy than those in the SH3/MD interface (19). Instead, the SH3/MD substitutions associate with hearing impairment: all patients carrying the mutations in the SH3/MD interface are expected to develop sensorineural deafness before 60 years of age, whereas the risk of developing nephropathy and cataract is low (19). Our patient carried a single nucleotide substitution in exon 2 of *MYH9* [c.130G > C (p.Ala44Pro)] located in the SH3/MD interface of the head domain, which has been previously described in one case (16); however, the natural history of the illness is unknown. Therefore, our patient should be carefully followed up especially for possible hearing loss than nephropathy. It is also important to instruct patients to avoid drugs that impair platelet and renal functions, including nonsteroidal anti-inflammatory drugs, antibiotics, and oncological drugs.

In conclusion, we describe a case of a girl with *MYH9*-RD carrying the c.130 G > C (p.Ala44Pro) mutation in *MYH9*; she was diagnosed with ITP. *MYH9*-RD can be misdiagnosed as ITP without a comprehensive approach involving blood smears or family history. Early recognition of inherited thrombocytopenia can prevent unnecessary diagnostic studies such as bone marrow aspiration and biopsy, and even adverse therapies with corticosteroids, immunosuppressive agents, and splenectomy.

## Data Availability

The original contributions presented in the study are publicly available. This data can be found here: https://www.ddbj.nig.ac.jp/index-e.html. Accession number: SAMD00776510.

## References

[1] Rodeghiero F Stasi R Gernsheimer T Michel M Provan D Arnold DM Standardization of terminology, definitions and outcome criteria in immune thrombocytopenic purpura of adults and children: report from an international working group. Blood. (2009) 113:2386–93. 10.1182/blood-2008-07-162503 19005182

[2] Balduini CL Pecci A Savoia A. Recent advances in the understanding and management of *MYH9*-related inherited thrombocytopenias. Br J Haematol. (2011) 154:161–74. 10.1111/j.1365-2141.2011.08716.x 21542825

[3] Favier R Feriel J Favier M Denoyelle F Martignetti JA. First successful use of eltrombopag before surgery in a child with *MYH9*-related thrombocytopenia. Pediatrics. (2013) 132:e793–5. 10.1542/peds.2012-3807 23940247

[4] Savoia A De Rocco D Pecci A. *MYH9* gene mutations associated with bleeding. Platelets. (2017) 28:312–5. 10.1080/09537104.2017.1294250 28368695

[5] Pecci A Ma X Savoia A Adelstein RS. *MYH9*: structure, functions and role of non-muscle myosin IIA in human disease. Gene. (2018) 664:152–67. 10.1016/j.gene.2018.04.048 29679756 PMC5970098

[6] Savoia A Pecci A Adam M Everman D Mirzaa G Pagon R editors (updated 2021). *MYH9*-related disease. GeneReviews®. In: Seattle (Wash.): University of Washington, Seattle (1993).

[7] Asensio-Juárez G Llorente-González C Vicente-Manzanares M. Linking the landscape of *MYH9*-related diseases to the molecular mechanisms that control non-muscle myosin II-A function in cells. Cells. (2020) 9:1458. 10.3390/cells9061458 32545517 PMC7348894

[8] Seri M Pecci A Di Bari F Cusano R Savino M Panza E *MYH9*-related disease: May–Hegglin anomaly, Sebastian syndrome, Fechtner syndrome, and Epstein syndrome are not distinct entities but represent a variable expression of a single illness. Medicine. (2003) 82:203–15. 10.1097/01.md.0000076006.64510.5c 12792306

[9] Hao J Kada A Kunishima S. Further classification of neutrophil non-muscle myosin heavy chain-IIA localization for efficient genetic diagnosis of *MYH9* disorders. Ann Hematol. (2018) 97(4):709–11. 10.1007/s00277-017-3195-3 29199357

[10] Noris P Biino G Pecci A Civaschi E Savoia A Seri M Platelet diameters in inherited thrombocytopenias: analysis of 376 patients with all known disorders. Blood. (2014a) 124:e4–10. 10.1182/blood-2014-03-564328 24990887 PMC4126341

[11] Noris P Klersy C Zecca M Arcaini L Pecci A Melazzini F Platelet size distinguishes between inherited macrothrombocytopenias and immune thrombocytopenia. J Thromb Haemost. (2009) 7:2131–6. 10.1111/j.1538-7836.2009.03614.x 19740094

[12] Kunishima S Matsushita T Kojima T Sako M Kimura F Jo EK Immunofluorescence analysis of neutrophil nonmuscle myosin heavy chain-A in *MYH9* disorders: association of subcellular localization with *MYH9* mutations. Lab Invest. (2003) 83:115–22. 10.1097/01.lab.0000050960.48774.17 12533692

[13] Savoia A De Rocco D Panza E Bozzi V Scandellari R Loffredo G Heavy chain myosin 9-related disease (*MYH9*-RD): neutrophil inclusions of myosin-9 as a pathognomonic sign of the disorder. Thromb Haemost. (2010) 103:826–32. 10.1160/TH09-08-0593 20174760

[14] Kitamura K Yoshida K Shiraishi Y Chiba K Tanaka H Furukawa K Normal neutrophil myosin IIA localization in an immunofluorescence analysis can rule out *MYH9* disorders. J Thromb Haemost. (2013) 11:2071–3. 10.1111/jth.12406 24106837

[15] Greinacher A Pecci A Kunishima S Althaus K Nurden P Balduini CL Diagnosis of inherited platelet disorders on a blood smear: a tool to facilitate worldwide diagnosis of platelet disorders. J Thromb Haemost. (2017) 15:1511–21. 10.1111/jth.13729 28457011

[16] Saposnik B Binard S Fenneteau O Nurden A Nurden P Hurtaud-Roux MF Mutation spectrum and genotype-phenotype correlations in a large French cohort of *MYH9*-related disorders. Mol Genet Genomic Med. (2014) 2:297–312. 10.1002/mgg3.68 25077172 PMC4113270

[17] Pecci A Panza E Pujol-Moix N Klersy C Di Bari F Bozzi V Position of nonmuscle myosin heavy chain IIA (NMMHC-IIA) mutations predicts the natural history of *MYH9*-related disease. Hum Mutat. (2008) 29:409–17. 10.1002/humu.20661 18059020

[18] Kahr WH Savoia A Pluthero FG Li L Christensen H De Rocco D Megakaryocyte and platelet abnormalities in a patient with a W33C mutation in the conserved SH3-like domain of myosin heavy chain IIA. Thromb Haemost. (2009) 102:1241–50. 10.1160/TH09-02-0119 19967157

[19] Pecci A Klersy C Gresele P Lee KJ De Rocco D Bozzi V *MYH9*-related disease: a novel prognostic model to predict the clinical evolution of the disease based on genotype-phenotype correlations. Hum Mutat. (2014) 35:236–47. 10.1002/humu.22476 24186861 PMC6233870

